# MRI noise and auditory health: Can one hundred scans be linked to hearing loss? The case of the Courtois NeuroMod project

**DOI:** 10.1371/journal.pone.0309513

**Published:** 2025-01-17

**Authors:** Eddy Fortier, Pierre Bellec, Julie A. Boyle, Adrian Fuente

**Affiliations:** 1 Département de Psychologie, Université de Montréal, Montréal, Québec, Canada; 2 Centre de Recherche de l’Institut Universitaire de Gériatrie de Montréal, Montréal, Québec, Canada; 3 École d’orthophonie et d’audiologie, Université de Montréal, Montréal, Québec, Canada; Universidade Federal de Sao Paulo/Escola Paulista de Medicina (Unifesp/epm), BRAZIL

## Abstract

Magnetic resonance imaging (MRI) is one of the most commonly used tools in neuroscience. However, it implies exposure to high noise levels. Exposure to noise can lead to temporary or permanent hearing loss, especially when the exposure is long and/or repeated. Little is known about the hearing risks for people undergoing several MRI examinations, especially in the context of longitudinal studies. The goal of this study was to assess the potential impact of repeated exposure to MRI noise on hearing in research participants undergoing dozens of MRI scans. This investigation was made possible thanks to an unprecedented intensive MRI research data collection effort (the Courtois NeuroMod project) where participants have been scanned weekly (up to twice a week), with the use of hearing protection, since 2018. Their hearing was tested periodically, over a period of 1.5 years. First, baseline pure-tone thresholds and distortion product otoacoustic emission (DPOAE) amplitudes were acquired before the beginning of this study. Hearing tests were then scheduled immediately before/immediately after a scan and with a delay of two to seven days after a scan. Pure-tone thresholds and DPOAE amplitudes showed no scanner noise impact right after the scan session when compared to the values acquired right before the scan session. Pure-tone thresholds and DPOAE amplitudes acquired in the delayed condition and compared to the baseline showed similar results. These results suggest an absence of impact from MRI noise exposure. Overall, our results show that an intensive longitudinal MRI study like the Courtois NeuroMod project likely does not cause hearing damage to participants when they properly utilize adequate hearing protection.

## Introduction

Modern research in human neuroscience and radiology relies heavily on magnetic resonance imaging (MRI). MRI is a versatile tool used for a variety of purposes by clinicians and researchers alike, including examination of post-stroke lesions [1, 2], reconstruction of white matter tracts [3–5] or observation of task-related brain activity through the hemodynamic response [6–8]. Since the introduction of MRI, more powerful scanners have periodically been introduced by manufacturers along with new acquisition sequences. Even though the scanner itself is very quiet, the activation of the acquisition sequence is an important source of high noise levels.

Each type of MRI acquisition having specific technical needs, multiple acquisition protocols have been developed, modified and optimized throughout the years. They all have specific parameters and electromagnetic properties. The choice of settings for some of those parameters has a major impact on the noise generated by this equipment [9–11], and thus, the level of risk to hearing varies from one sequence to another. For example, in their study on the effects of MRI noise exposure on the auditory system, Radomskij et al. [12] measured peak noise levels between 122 and 131 dB SPL for the sequences they used. Given these noise levels and how hazardous they can be to hearing, even if the exposure is short, England’s health protection agency strongly recommends using MRI-compatible hearing protection devices [13].

Exposure to these high levels of noise is even more of a factor for studies that require participants to be scanned multiple times in a short period and/or scanned repeatedly for a long duration (i.e., years). Although these types of study, producing what is known as deep and dense datasets [14], have a high value to neuroscientists [15, 16] and aid our understanding of the human brain [17, 18], they may raise some concerns over these participants’ hearing health. Jin et al. [19] showed that both prolonged exposure and elevated levels of noise in the MRI scanner can be linked to temporary hearing threshold shifts, even when wearing hearing protection devices. Similarly, Radomskij et al. [12] reported a significant effect of exposure to MRI noise on cochlear function, despite the use of earplugs. These findings are not surprising given that the recommended daily noise exposure dose can be reached in only a few minutes when exposed to MRI noise. According to the United States National Institute for Occupational Safety and Health (NIOSH) [20], the daily recommended noise exposure limit is 85 dBA as an 8-hour time-weighted average (TWA). Therefore, exposures to noise levels higher than 85 dBA imply shorter maximum daily doses. For example, the maximum daily dose for exposure to a 100-dBA noise is only 15 minutes [20]. On the other side of the Atlantic ocean, the European Agency for Safety and Health at Work’s Directive 2003/10/EC requires that hearing protections are made available to workers when the 8-hour TWA noise exposure exceeds 80 dBA and renders their use mandatory when it exceeds 85 dBA [21].

Noise exposure can induce either temporary or permanent changes in hearing thresholds [22]. The recovery time from temporary threshold shifts (TTSs) is variable and depends on a number of factors directly associated with the noise exposure, such as the frequency spectrum of the noise, the temporal characteristics (continuous versus impulsive) and the duration of the exposure [23, 24]. In addition, the presence of an effective quiet period after the exposure, its quality (ambient noise level) and length influence the recovery time after a noise exposure [23]. TTSs indicate that the noise exposure was hazardous to hearing [25, 26]. Recurrent TTSs can lead to permanent threshold shifts, which may subsequently lead to hearing loss (i.e., hearing thresholds poorer than 20 dB HL [27, 28]). Conventional pure-tone audiometry (250 Hz– 8 kHz) is currently used in hearing conservation programs to monitor hearing in noise-exposed workers [20]. However, extended high-frequency pure-tone audiometry (9–20 kHz) is recommended to detect early signs of noise-induced hearing loss (NIHL) [29]. In addition to pure-tone audiometry, otoacoustic emissions (OAEs) can be used with the aim to evaluate the function of outer hair cells in the cochlea. Outer hair cells are one of the main targets of noise exposure, and thus changes in OAEs can precede changes in conventional pure-tone thresholds [30, 31]. Therefore, OAEs have been suggested to be used for the early detection of noise-induced hearing loss and/or for monitoring hearing in people exposed to noise [32, 33].

Little is known about the impact of cumulative MRI noise exposure on individuals who are periodically scanned and who utilize hearing protection while being tested. This issue is important for deep and dense dataset projects in general, and is particularly relevant for the participants of the Courtois NeuroMod project, an MRI research data collection effort of unprecedented scale that spanned 5 years and involved weekly MRI scans [34]. Thus, the goal of this study was to evaluate possible impact on hearing induced by repeated MRI scanning on the Courtois NeuroMod participants (n = 6). If present, such an impact would indicate hazardous noise exposure levels, even with the use of hearing protection. If absent, this work would document cases where MRI noise did not impact participants’ hearing with the largest amount of exposure to date, even though these observations may not generalize outside of the Courtois NeuroMod experimental setup. We designed a hearing test protocol to capture both clinical and subclinical changes over time. Specifically, the Courtois NeuroMod research participants were tested for temporary and permanent changes in hearing outcomes with clinical (pure-tone audiometry: 250 Hz– 8 kHz) and subclinical (pure-tone audiometry: 9 kHz– 20 kHz and distortion product OAEs (DPOAEs)) auditory tests. To detect possible temporary changes in hearing outcomes, participants were evaluated immediately before and immediately after the scanning sessions. For permanent changes in hearing outcomes, participants were evaluated between 48 hours and seven days after a scanning session. In this case, hearing outcomes (i.e., pure-tone thresholds and DPOAE amplitudes) were compared with the baseline values that were obtained before we started the hearing health monitoring project.

## Methods

### The Courtois NeuroMod project

This research project is part of a larger initiative called the Courtois NeuroMod project–CNeuroMod [34]. The CNeuroMod project is an intensive MRI scanning platform designed to acquire massive amounts of brain imaging data on a limited number of participants performing a wide range of tasks. Participants take part in scanning sessions lasting between 1.5 and 3 hours per week over a period of six years. This period started between the fall of 2018 and summer of 2019 (variable for each participant). Such an intensive scanning schedule raises health and safety concerns, including the hearing health of the participants [19]. Thus, a subcomponent of the Courtois NeuroMod project was to monitor the participants’ hearing health for the duration of the study.

### Participants

The participant group included three self-reported women and three self-reported men who all indicated being right-handed at the beginning of the CNeuroMod project. Their age ranged from 33 to 49 years (mean = 42.58 years, SD = 6.14 years) when we acquired the baseline values for the hearing tests used in the monitoring protocol. Their age ranged from 33 to 50 years (mean = 43.38, SD = 6.43 years) at the time of their last test session. The time interval between the baseline session and the last test session varied between 1.15 and 16.11 months (mean = 9.55 months, SD = 5.66 months). All participants gave their written consent to participate in the CNeuroMod project, which was approved by the local ethics committee (project CER VN 18-19-22 of the ageing and neuroimaging research ethics committee at the Centre de recherche de l’Institut universitaire de gériatrie de Montréal (CRIUGM)). The exclusion criteria included visual and hearing impairments that would prevent participants from being able to see and hear the presented stimuli and the standard MRI and magnetoencephalography exclusion criteria (presence of magnetic metal pieces in the body: pacemaker, aneurysm clips, metallic stents, shrapnel/bullet fragments, certain orthopedic implants, etc.). Since this study is done as part of a larger research project, the sole inclusion criterion for the present study was to be enrolled in the CNeuroMod research project. One participant (sub-04) temporarily suspended scanning during the course of the hearing test protocol. Therefore, we stopped monitoring their hearing, and thus their dataset is smaller than that of the other participants. Another participant (sub-05) had a pre-existing hearing impairment and no OAE response in the left ear (see S1 Fig). To make sure that the scanning sessions were not causing further impairment, we subjected this participant to an intensive five-week pure-tone audiometry protocol where they were tested before and after each scan session, making their dataset larger than that of the other participants. Lastly, participant schedules and availability were important variables in our study, resulting in not all subjects having the same number of hearing test sessions, and thus, not all subjects have an equal number of sessions for each test condition.

### Protocol design

To accomplish the aim of the study, three experimental conditions were designed (Table 1): two conditions to detect possible temporary changes in hearing outcomes (pre-scan and post-scan: short-term observations), and one condition to detect possible permanent changes in hearing outcomes (delayed post-scan session: long-term observation). Therefore, each observation comprises two test sessions, each one of them being identified by an identifier formed by the abbreviation “ses-” and a two-digit number (e.g., ses-05). The goal of the short-term observations was to see if the noise exposure associated with a given scanning session had an adverse effect on the auditory system. It is important to note that such changes may be temporary or permanent. However, obtaining hearing outcomes immediately before and after the noise exposure makes it possible to determine if that particular noise exposure was harmful to the auditory system. The goal of the delayed observation was to see if the cumulative scanning sessions had an enduring impact on hearing. This was done by comparing hearing test results obtained in a test session carried out between 48 hours and 7 days after a scanning session and those obtained at baseline (beginning of this research project: January–February 2021). In addition, this condition was used to determine whether any changes in hearing outcomes observed in the previous conditions (immediately before/immediately after the scanning session) remained. If that was the case, then such changes were considered permanent rather than temporary.

**Table 1:** Hearing test distribution in three experimental conditions.

Condition type	Condition number		Otoscopy	Tymp	Reflex	PTA	DPOAE
Baseline			X	X	X	X	X
Short-term	1	Pre-scan	X	X	X	X	
		Post-scan				X	
	2	Pre-scan	X	X	X		X
		Post-scan					X
Long-term	3	Delayed	X	X	X	X	X

Tymp: Tympanometry. Reflex: Stapedial reflex. PTA: Pure-tone audiometry. DPOAE: Distortion product otoacoustic emissions.

### Hearing tests

#### Outer and middle ear

Prior to each test session, we conducted a routine check to ensure the absence of external and middle-ear problems. First, a visual otoscopic examination was performed using a Medical Pro otoscope to ensure the absence of obstructing debris in the external auditory canal and the integrity of the tympanic membrane. Then, to assess the mobility of the tympanic-membrane-ossicles system, tympanograms were obtained using an 85 dB SPL probe tone at a frequency of 226 Hz (MADSEN Zodiac Type 1096 SA, GN Otometrics A/S, now Natus Medical Inc.). In addition, ipsilateral stapedial reflexes at 0.5 kHz, 1 kHz, 2 kHz, 4 kHz and with broadband noise (400–4000 Hz) were acquired.

#### Pure-tone audiometry

Air conduction pure-tone thresholds were obtained using the Otometrics OTOSuite software (version 4.84.0.61) and a MADSEN Astera2 Type 1066 clinical audiometer (GN Otometrics A/S, now Natus Medical Inc.). Both the standard frequency range (0.25, 0.5, 1, 2, 3, 4, 6 and 8 kHz) and the extended high frequency range (9, 10, 11.2, 12.5, 14, 16, 18 and 20 kHz) were tested using 5-dB-HL steps. Pure-tone stimuli were first presented to the participants using Otometrics insert earphones with 3M E-A-RLink type 3A foam ear tips for the standard frequency range and then using Sennheiser HDA 300 headphones for the extended high frequency range. Participants were instructed to press a single-button response box as soon as they thought they heard a sound. First, the right-ear thresholds were obtained, then the left-ear thresholds. The same person (a trained clinical audiologist) tested all participants across the sessions using a modified Hughson and Westlake [35] procedure as described by Carhart and Jerger [36]. Calibration of the stimuli presentation equipment according to ANSI/ASA S3.6–2010 [37] was done before the beginning of the project.

Single-frequency measured differences were divided into three categories using a strict threshold generalized to all tested frequencies [38] and a more widely accepted threshold [39]. This decision was based on the following reasons: 1) we had an insufficient amount of repetitions of each test condition to properly apply statistical analyses and corrections for multiple comparisons, 2) the lack of consensus in the field regarding how to assess the significance of test-retest differences in pure-tone audiometry [20, 39–46], and 3) the variable per-frequency significant threshold shift values found in the literature [38, 47, 48]. Therefore, threshold shifts were considered negligible and non-significant if they were showing improvements or deteriorations no greater than 5 dB HL (*threshold shift* ∈ [-∞, 5]) [38]. Threshold shifts were considered significant but mild if they were greater than 5 dB HL but smaller than 20 dB HL (*threshold shift* ∈ [5, 20]). Threshold shifts were considered significant and severe if they showed deteriorations of 20 dB HL or more (*threshold shift* ∈ [20, ∞]) [39]. S1 Table displays a brief comparison of the different pure-tone significant-threshold-shift criteria mentioned here. Note that none of the subjects presented with a measurable hearing threshold at 18 and 20 kHz in all testing sessions.

#### Otoacoustic emissions

The function of the outer hair cells of the cochlea was evaluated through the use of DPOAEs. DPOAE amplitudes were obtained by the simultaneous presentation of two primary frequencies using a repeating downward presentation cycle. An F2/F1 ratio of 1.22 and presentation levels L1/L2 of 65/55 dB SPL were used. The F2 frequencies included 1, 1.5, 2, 3, 4, 6, 8 and 10 kHz. DPOAE tests were administered using the ILOv6 software (version 6.41.27.33), an Echoport ILO292 USB-II stimuli presentation device (version 2.001J) and a UGD probe (Otodynamics Ltd.). The measured DPOAE amplitudes (in dB SPL) had to exceed the noise floor by more than two standard deviations to be considered present. Therefore, values are missing in the OAE figures for some participants due to DPOAE responses that did not exceed this threshold during pre-scan, post-scan, baseline and/or delayed test session(s). These missing values are all located in the extreme parts of the tested spectrum (for F2 = 1 kHz and 1.5 kHz or for F2 = 8 kHz and 10 kHz). To evaluate the significance of DPOAE test-retest differences, the Keppler at al. [49] minimal detectable difference (95%) criteria for a 60-minute delay (short-term observation; 1 kHz: 3.78 dB, 1.4 kHz: 2.90 dB, 2 kHz: 2.06 dB, 2.8 kHz: 2.09 dB, 4 kHz: 1.83 dB, 6 kHz: 1.58 dB, 8 kHz: 2.80 dB) and a 7-day delay (long-term observation; 1 kHz: 4.05 dB, 1.4 kHz: 3.52 dB, 2 kHz: 2.69 dB, 2.8 kHz: 1.97 dB, 4 kHz: 2.55 dB, 6 kHz: 2.53 dB, 8 kHz: 4.43 dB) were used because they were the closest to our time intervals between two tests. Approximations had to be made since the criteria had values for 1.0, 1.4, 2.0, 2.8, 4.0, 6.0 and 8.0 kHz. Therefore, we used the threshold value established at 1.4 kHz for 1.5 kHz, the value at 2.8 kHz for 3.0 kHz and the value at 8 kHz for 8 and 10 kHz. Calibration of the stimuli presentation equipment was done before the beginning of the project.

Pure-tone audiometry and DPOAE tests were administered to the participants while they were seated on a chair inside a GENIE Series model GN-17 soundproof booth (GN Otometrics A/S, now Natus Medical Inc.).

#### Detection of clinical and subclinical changes

A clinical impact was considered present when a standard pure-tone (250 Hz– 8 kHz) threshold shift would reach the mild threshold shift range (see Pure-tone audiometry section). A subclinical impact was considered present when the global portrait given by the DPOAE amplitudes and extended high frequency pure-tone thresholds showed significant shifts (DPOAE: amplitude decrease, see Otoacoustic emissions section; extended pure-tone threshold: mild threshold increase, see Pure-tone audiometry section) for both these tests. It was also expected that these changes would be consistent (through ears, sessions and participants) [31] and would start from the highest end of the hearing spectrum, potentially progressing toward the lower frequencies as subclinical changes evolve into clinical changes [29, 50].

### Experimental conditions

Hearing sessions in the short-term observations were added on top of the scanning session and were quite demanding for participants. To lighten the test load for the participants, this condition was split into two sessions based on the tests used: PTA and DPOAE (see Table 1, conditions #1 and #2). The test condition carried out between 48 hours and 7 days after a scanning session included all tests (see Table 1, condition #3).

### MRI acquisition

Hearing outcomes associated with noise exposures from two types of MRI acquisition sessions (i.e., anatomical and functional) were investigated in this study. This is required because the different MRI acquisition sequences used in the anatomical and functional sessions could also be linked with different noise exposures in terms of amplitude and spectrum. The MRI scanner used is a 3 T Siemens Prisma Fit scanner located at Unité de Neuroimagerie Fonctionnelle (CRIUGM, Montréal, Canada). This scanner is equipped with a 2-channel transmit body coil and the data acquisition is done using a 64-channel receive head/neck coil antenna. Detailed information on scanning sequences and parameters is available at this address: https://docs.cneuromod.ca/en/latest/MRI.html. Total scanning time for each of the anatomical sessions is approximately 1 hour and 5 minutes and scanning time for each of the functional sessions is between 45 minutes and 2 hours and 30 minutes.

#### MRI sequences’ noise levels

The different MRI acquisition sequences used during the scanning sessions can be linked to different noise exposures. For safety reasons linked to the magnetic properties of the MRI scanner, noise levels associated with each of the scanner sequences were acquired with a sound level meter (Piccolo sound level meter model SLM-P1, Soft dB; sampling frequency: 1 Hz) on a tripod stand placed just outside of the MRI scanner room. The distance between the entrance of the scanner’s bore and the sound level meter was 5.2 meters (~17 feet) at an angle of approximately 40–45 degrees to the bore axis. It is important to note here that the actual noise levels experienced by research participants are considerably higher than what we measured due to their closer proximity to the noise source and the narrowness of the bore (60 centimeters). Table 2 presents the sound levels associated with each of the MRI sequences used by the CNeuroMod project. Noise levels were measured for the shortest of two durations: approximately one minute, or the whole sequence’s duration (for sequences that are activated for less than one minute: localizer sequences (19 seconds), scout sequence (14 seconds), and AP phase encoding sequence (15 seconds)).

**Table 2:** Sound levels associated with the MRI acquisition sequences used by the Courtois NeuroMod project.

Sequences		Sound levels (dBA)			
		Mean	SD	Min	Max
**Common sequence**					
	localizer (brain) sequence	73.08	2.06	70.5	77.2
**Anatomical sessions’ sequences**					
*Brain sequences*					
	T1-weighted MPRAGE 3D sagittal sequence	71.88	0.70	70.5	73.6
	T2-weighted FSE (SPACE) 3D sagittal sequence	71.38	8.65	59.8	81.8
	diffusion-weighted 2D axial sequence	80.91	0.53	79.9	81.6
	gradient-echo magnetization-transfer 3D sequence	69.00	0.52	67.9	70.1
	gradient-echo proton density 3D sequence	69.12	0.78	67.6	70.5
	gradient-echo T1-weighted 3D sequence	70.83	0.69	69.4	72.2
	MP2RAGE 3D sequence	66.69	6.55	59.6	73.7
	susceptibility-weighted 3D sequence	71.89	0.37	71.1	72.7
*Spinal cord sequences*					
	localizer (spinal cord) sequence	70.61	1.19	68.3	73.2
	T2-weighted 3D sagittal sequence	72.47	6.26	62.8	78.7
	diffusion-weighted 2D axial sequence	74.33	1.38	72.0	76.6
	gradient-echo magnetization-transfer 3D axial sequence	71.71	0.67	70.9	73.1
	gradient-echo T1-weighted 3D axial sequence	73.69	0.73	72.8	75.3
	gradient-echo ME	71.76	1.28	69.0	74.0
**Functional sessions’ sequences**					
	Scout sequence	75.16	1.29	71.8	76.8
	AP phase encoding sequence	80.57	0.26	79.7	80.7
	accelerated simultaneous multi-slice, gradient echo-planar imaging sequence [51]	81.35	0.43	80.2	82.0
	accelerated simultaneous multi-slice, gradient echo-planar imaging sequence [51]: Emotions’ dataset variant	80.09	0.19	79.6	80.3

SD: Standard deviation. Min: Minimum. Max: Maximum.

#### Hearing protection

The use of hearing protection in the MRI scanner is strongly recommended, especially when auditory stimuli are involved in the scanning session [52]. The hearing protection used by the participants during the scanning sessions was a combination of devices that evolved since the beginning of the CNeuroMod project. The purpose of these iterations was to constantly try to provide the best level of protection possible for the participants’ hearing while allowing the presentation of audio stimuli. At the beginning of the project, they consisted of a combination of in-house modified commercial earmuffs (Leightning L2F Premium Folding Earmuff, Stanley Black & Decker Inc.; Pre-modification advertised Noise Reduction Rating: 27 dB), and an S15 MRI-compatible earphone system (Sensimetrics Corporation) coupled with standard-size, original-style Comply disposable canal tips (Hearing Components, Inc.; Advertised Noise Reduction Rating: 29 dB). The S15 is a commonly used earphone system [53] used to present high-quality auditory stimuli to research participants. Modification of the commercial earmuffs was necessary to allow them to fit inside the head coil antenna. Unfortunately, these modifications likely negatively influenced their effectiveness, and more importantly, generated pressure points that caused major discomfort for some of the participants. Moreover, the chosen length and size of the canal tips were not optimal for everyone’s auditory canal, leading to potential variability in the quality of the fit and level of protection. These problems were iteratively addressed over the course of the study, and the last evolution of the device combination includes participant-specific-size Comply disposable canal tips, as well as headphone replacement memory foam rings (Brainwavz Audio).

## Results

### Short-term observations

**Fig 1:**
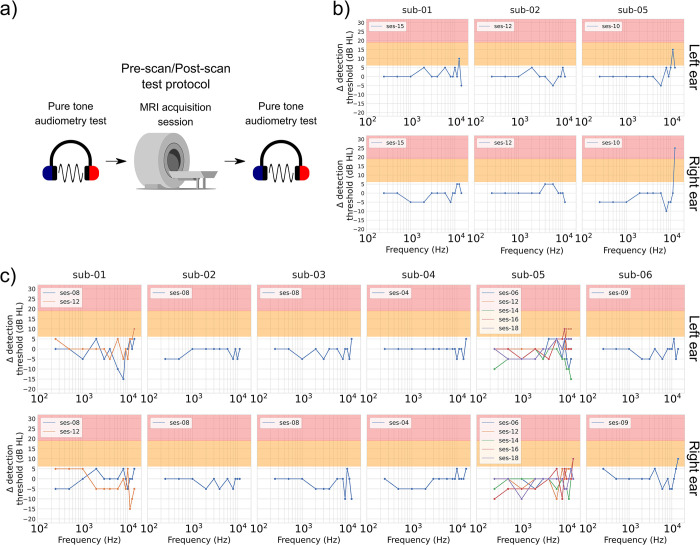
Pure-tone audiometric threshold differences (in dB HL), post-scan minus pre-scan, as a function of the pure-tone frequency. a) presents a schematic description of the test session progress. Results are presented for b) anatomical scans and c) functional scans, as these sequences do not generate similar noises in terms of both amplitude and frequency spectrum. Pure-tone threshold results are presented for both standard (0.25, 0.5, 1, 2, 3, 4, 6 and 8 kHz) and extended high frequency (9, 10, 11.2, 12.5, 14, 16, 18, and 20 kHz) ranges. For each participant, graphs on the top row are for the left ear, while graphs on the bottom row are for the right ear. Worsening hearing thresholds of a mild degree (> 5 but < 20 dB HL) are highlighted with an orange background, while severe increases (≥ 20 dB HL) are highlighted with a red background. When multiple observations have been conducted per participant, each line represents a different observation. Missing values in the graph indicate that the participant did not give any behavioral response at that frequency pre- and/or post-scan. Original images in panel a) were adapted from vectorportal.com (MRI scanner; CC BY 4.0 license), svgrepo.com (headphones; CC0 license), and commons.wikimedia.org (sinus wave; released into the public domain by Mikael Häggström).

**Fig 2:**
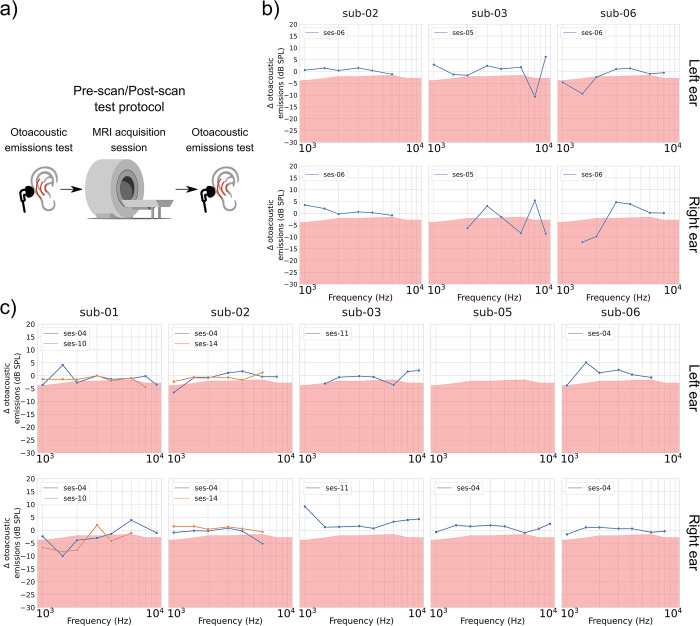
DPOAE differences (in dB SPL), post-scan minus pre-scan, as a function of the F2 frequency. a) presents a schematic description of the test session progress. Results are presented for b) anatomical scans and c) functional scans. DPOAE amplitudes are presented for F2 = 1, 1.5, 2, 3, 4, 6, 8 and 10 kHz. For each participant, graphs on the top row are for the left ear, while graphs on the bottom row are for the right ear. Significant amplitude decreases [49] are highlighted with a red background. When multiple observations have been conducted per participant, each line represents a different observation. Missing values in the graph indicate that the participant’s DPOAE amplitude did not exceed the established noise floor threshold (see Methods, Otoacoustic emissions section) for that F2 frequency pre- and/or post-scan. Original images in panel a) were adapted from vectorportal.com (MRI scanner; CC BY 4.0 license), svgrepo.com (earphones; CC0 license), and freesvg.org (ear; public domain).

The goal of the short-term observation was to monitor potential temporary effects of scanner noise on the participants’ hearing. To do so, hearing tests supplying clinical (standard pure-tone audiometry) and subclinical (extended high frequency pure-tone audiometry and DPOAE) information on the participants’ hearing health were performed immediately before and after a scanning session. Three participants were tested before/after an anatomical scanning session (pure-tone: sub-01, 02 and 05; DPOAE: sub-02, 03 and 06). Five participants’ pure-tone thresholds were tested once or twice before/after a functional scanning session (sub-01, 02, 03, 04 and 06). Sub-05 pure-tone thresholds were tested more extensively (see Participants section). Five participants’ DPOAE amplitudes were tested once or twice before/after a functional scanning session (sub-01, 02, 03, 05 and 06). Figs 1 and 2 display the results for the two outcomes: pure-tone thresholds (Fig 1) and DPOAE amplitudes (Fig 2).

#### Clinical outcome shows no short-term impact of MRI noise exposure

Hearing thresholds acquired right after a scan session were compared to hearing thresholds acquired right before that same scan session (post-scan value–pre-scan value). In Fig 1, a mild increase in hearing thresholds is highlighted with an orange background, while a severe increase of hearing thresholds is highlighted with a red background (see Methods, Pure-tone audiometry section). Using this significance criteria, standard frequencies (250 Hz– 8 kHz) showed no significant threshold increase (difference greater than 5 dB HL) for both anatomical and functional acquisition sequences.

#### Subclinical outcomes were variable, and were not indicative of short-term impact of MRI noise exposure

DPOAE amplitudes (in dB SPL) measured right after a scan session were compared to the amplitudes measured right before that same scan session (post-scan value–pre-scan value). In Fig 2, a significant DPOAE amplitude decrease (see Methods, Otoacoustic emissions section) is highlighted with a red background. For the anatomical scans (Fig 2, panel b), some changes were observed, but were inconsistent across subjects. For example, sub-03 and sub-06 exhibited significant DPOAE amplitude reductions in both ears while sub-02 did not show amplitude reduction. Changes were also inconsistent in terms of the F2 involved. For example, changes were detected for different high F2 frequencies for the two ears of sub-03. Amplitude reductions in sub-06 were detected at low F2 frequencies. These observations were thus not indicative of subclinical hearing damages, but rather of test-retest variability.

Regarding the results after functional scan sessions (Fig 2, panel c), changes were also observed inconsistently across subjects and frequencies. Specifically, sub-01 showed significant DPOAE amplitude reductions while amplitude reductions were minimal or non-existent for sub-02, sub-03, sub-05 and sub-06. Changes observed in sub-01 were mainly in the right ear and more pronounced for F2s in the low frequency spectrum. On the other hand, some DPOAE amplitudes in some subjects actually improved (i.e., higher amplitudes) after a functional scan session. These results were thus again not suggestive of subclinical injury.

Extended high frequency pure-tone thresholds (9 kHz– 20 kHz) followed a similar behavior to DPOAE amplitudes, with inconsistent changes detected across ears, sessions and subjects. After anatomical scanning sessions (Fig 1, panel b), sub-01 and sub-05 exhibited threshold increases. After functional scanning sessions (Fig 1, panel c), sub-01, sub-05 and sub-06 showed threshold increases. While sub-01 and sub-05 were tested more than once, changes at a given frequency were variable between sessions. Significant improvements in hearing thresholds (i.e., negative threshold shifts) were also observed in some participants (sub-01, 03 and 05) after scanning sessions, particularly after functional scanning sessions. These results were thus not suggestive of subclinical injury, but rather of a high test-retest variability.

In summary, DPOAE amplitude profiles were inconsistent (between ears, sessions and/or participants) and the extended high frequency pure-tone thresholds exhibited high test-retest variability. Taken together, these results were not indicative of a temporary subclinical effect of MRI noise exposure on the participants’ hearing.

### Delayed observations

**Fig 3:**
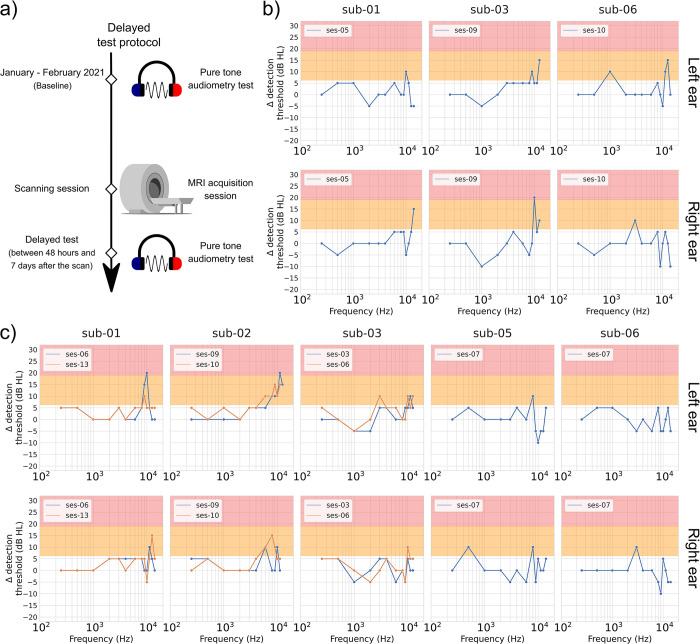
Pure-tone audiometric threshold differences (in dB HL), delayed observation minus baseline, as a function of the pure-tone frequency. a) presents a schematic description of the test session progress. Results are presented for b) anatomical scans and c) functional scans. Pure-tone threshold results are presented for both standard (0.25, 0.5, 1, 2, 3, 4, 6 and 8 kHz) and extended high frequency (9, 10, 11.2, 12.5, 14, 16, 18, and 20 kHz) ranges. For each participant, graphs on the top row are for the left ear, while graphs on the bottom row are for the right ear. When multiple observations have been conducted per participant, each line represents a different observation. Worsening hearing thresholds of a mild degree (> 5 but < 20 dB HL) are highlighted with an orange background, while severe increases (≥ 20 dB HL) are highlighted with a red background. Missing values in the graph indicate that the participant did not give any behavioral response at that frequency during the delayed observation and/or the baseline. Original images in panel a) were adapted from vectorportal.com (MRI scanner; CC BY 4.0 license), svgrepo.com (headphones; CC0 license), and commons.wikimedia.org (sinus wave; released into the public domain by Mikael Häggström).

**Fig 4:**
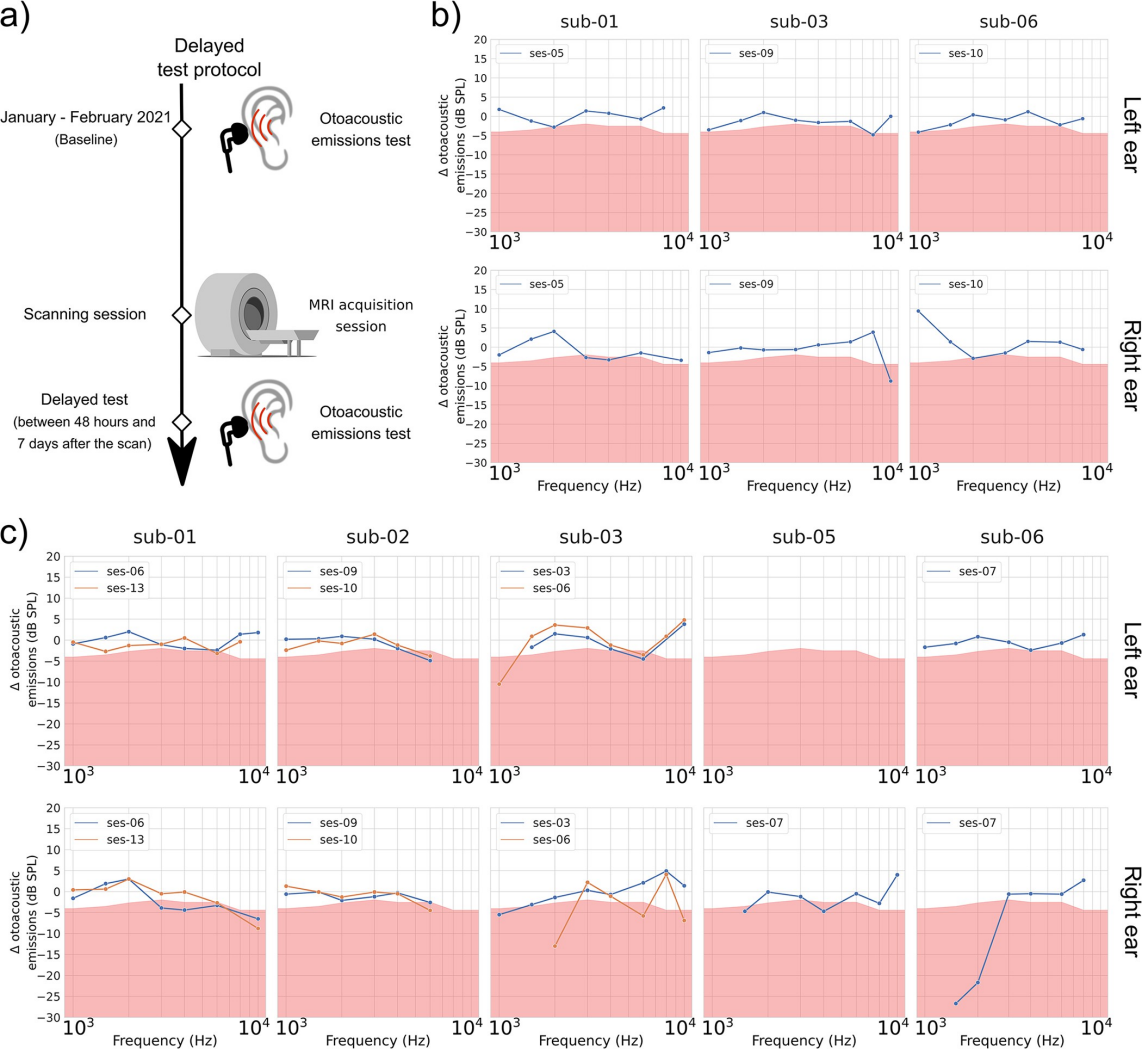
DPOAE differences (in dB SPL), delayed observation minus baseline, as a function of the F2 frequency. a) presents a schematic description of the test session progress. Results are presented for b) anatomical scans and c) functional scans. DPOAE amplitudes are presented for F2 = 1, 1.5, 2, 3, 4, 6, 8 and 10 kHz. For each participant, graphs on the top row are for the left ear, while graphs on the bottom row are for the right ear. Significant amplitude decreases [49] are highlighted with a red background. When multiple observations have been conducted per participant, each line represents a different observation. Missing values in the graph indicate that the participant’s OAE response did not exceed the established noise floor threshold (see Methods, Otoacoustic emissions section) for that F2 frequency during the delayed observation and/or the baseline. Original images in panel a) were adapted from vectorportal.com (MRI scanner; CC BY 4.0 license), svgrepo.com (earphones; CC0 license), and freesvg.org (ear; public domain).

The goal of the delayed observation was to see if the cumulative scanning sessions have an enduring impact on hearing. To do so, hearing tests supplying clinical (standard pure-tone audiometry) and subclinical (extended high frequency pure-tone audiometry and DPOAE) information on the participants’ hearing health were performed in a time frame between 48 hours and 7 days (168 hours; mean = 139.34 hours, SD = 46.92 hours) after a scanning session along the study period. Three participants were tested during this time interval after an anatomical scanning session, and five participants were tested after a functional scanning session. These observations were then compared to the baseline pure-tone thresholds and DPOAE amplitudes acquired at the beginning of the study (January–February 2021). Figs 3 and 4 display the results for the two outcomes: pure-tone thresholds (Fig 3) and DPOAE amplitudes (Fig 4). Table 3 displays the time interval between the baseline session and each of the delayed observations.

**Table 3:** Time intervals between the baseline session and the delayed observations.

Participant	Session ID	Delay (from the baseline)
Sub-01	ses-05	2 months, 3 days
	ses-06	3 months, 18 days
	ses-13	12 months, 23 days
Sub-02	ses-09	4 months, 4 days
	ses-10	7 months, 24 days
Sub-03	ses-03	1 month, 3 days
	ses-06	2 months, 15 days
	ses-09	8 months, 21 days
Sub-05	ses-07	2 months, 21 days
Sub-06	ses-07	3 months, 0 day
	ses-10	8 months, 1 day

#### Clinical outcome shows inconsistent long-term impact of MRI noise exposure

Hearing thresholds acquired during the delayed observations were compared to the baseline thresholds (test session values–baseline values). Using the significance criteria established for this study (see Methods, Pure-tone audiometry section) in connection with anatomical scanning sessions (Fig 3, panel b), two out of the three tested participants (sub-01 and 03) showed no significant increment in hearing thresholds for the standard audiometric frequencies (250 Hz– 8 kHz). The third one (sub-06) showed limited and isolated threshold increases. When looking at test sessions linked to functional scans (Fig 3, panel c), four out of the five participants (sub-02, 03, 05 and 06) showed mild increments for at least one tested frequency in the standard range. Detected clinical changes involved different frequencies across participants, as well as different frequencies between the left and right ears. In summary, long-term clinical changes were detected, although these changes were inconsistent.

#### Subclinical outcomes show inconsistent long-term impact

DPOAE amplitudes measured during the delayed observations were compared to the reference values acquired during the baseline session (test session values–baseline values). Based on the test-retest variability criterion used for this study (see Methods, Otoacoustic emissions section), DPOAE amplitudes at some frequencies showed significant reductions in the delayed observations when compared with the baseline. However, such reductions were mainly in isolated frequencies. Individual data shows that sub-01 exhibited DPOAE amplitude reductions up to around 9 dB SPL in the right ear (for F2 = 10 kHz). Sub-03 showed a maximum reduction of 10.5 dB SPL for F2 = 1 kHz (left ear) and 13 dB SPL for F2 = 2 kHz (right ear). Finally, sub-06 showed maximum DPOAE amplitude reductions in the right ear of 26.7 and 21.7 dB SPL for F2 = 1.5 and 2 kHz, respectively. All other DPOAE frequencies either showed amplitude reductions that did not exceed 5 dB SPL or did not show amplitude reductions in both ears. It should also be noted that DPOAE amplitudes for some F2 frequencies in some subjects significantly improved (i.e., higher amplitudes) after a scan session.

Regarding the extended high frequencies (9–20 kHz), mild significant increments in hearing thresholds were observed in four of the five tested subjects in at least one ear (see Fig 3). In addition, 20-dB-HL increments in hearing threshold were observed with sub-01, sub-02, and sub-03. However, for sub-01 and 02, hearing thresholds in these frequencies improved in subsequent testing sessions and fell within the range of mild threshold shift (sub-02) or within the test-retest variability (sub-01). It can also be observed in Fig 3 that delayed observations showed better hearing thresholds (i.e., lower hearing thresholds) in some follow-up sessions than during the baseline. All such improvements in hearing thresholds did not exceed 10 dB HL.

In summary, the DPOAE delayed observations present two types of amplitude patterns: a more unstable pattern with both significant amplitude increases and decreases, and a more stable one closer to the amplitude variation’s neutral point (a 0-dB-SPL difference). These inconsistent DPOAE amplitude patterns (between ears, sessions and/or participants), when combined with the also inconsistent extended high frequencies’ patterns of threshold gains/losses and apparent greater test-retest variability, show no consistent long-term subclinical effect of the exposure to the MRI scanner noise.

## Discussion

### Overview of the results

The aim of this study was to evaluate possible clinical and subclinical effects on hearing of intensive exposure to scanner noise. To investigate this, participants got their hearing tested immediately before and after scan sessions (to test short-term effects) as well as after a period of at least 48 hours (to test long-term effects).

Overall, observations on short-term effects showed no significant clinical impact of the exposure to the scanner noise on standard pure-tone thresholds (250 Hz– 8 kHz). Observations on short-term subclinical changes, combining DPOAE amplitudes and extended high frequency pure-tone thresholds (9 kHz– 20 kHz), showed no consistent effect of the noise exposure.

The delayed observations showed inconsistent effect of the noise exposure on both the clinical (standard pure-tone thresholds) and the subclinical observations (DPOAE amplitudes and extended high frequency pure-tone thresholds combination).

### Temporary changes in hearing outcomes

As previously mentioned, the clinical pure-tone thresholds (250 Hz– 8 kHz) obtained in this protocol did not show a short-term impact of scanner noise exposure since most of the threshold variations measured for this outcome during this period fell within the test-retest variability criteria or presented an improvement after the scan.

The subclinical short-term effects were evaluated with the combination of two hearing tests: the DPOAE amplitudes and the extended high frequency pure-tone thresholds (9 kHz– 20 kHz). Since the DPOAE test is an objective evaluation that does not require any form of behavioral response from participants, the level of fatigue is not likely to have a significant impact on the outcome. The inconsistent results obtained for this test seem to relate to a hypothesis raised by Govindaraju et al. [54] when they reported a case study of a subject who complained about a blocked sensation followed by tinnitus in his right ear, but no subjective hearing loss, following a 41-minute anatomical scan of his lower back. The subject used foam plugs during the scan, but a pure-tone audiometry test performed the next day revealed sensorineural hearing loss on the right side, supporting the subject’s complaint and showing a possibly permanent impact of the exposure to MRI noise. The lateralized impact presented in that report could suggest an improperly placed earplug in the affected ear, but the absence of a pre-scan reference prevents the complete exclusion of possible pre-existing, undocumented hearing loss. They hypothesized that a lateralized incorrect use of hearing protection could possibly have decreased its efficiency, highlighting the importance of the proper placement and adjustment of hearing protection devices. In our study, the DPOAE amplitudes showed some impacts for some participants, but the lack of between-ears and between-sessions consistencies seem to also indicate a hearing protection device placement issue that could momentarily have affected the results for a specific ear during a specific session. A less than optimal placement of the hearing protection equipment or a problem in the fitting of the foam canal tip due to an inadequate choice of size for the participant’s auditory canal can probably be linked to some of these observations through a diminished effectiveness of the noise reduction. Moreover, the different patterns observed between the two ears that three of the participants showed for this type of observation tend to support this idea that on one side, the protection must have been properly installed, while the other side was not sealed properly, affecting the sound spectrum reaching the ear. Yildirim et al. [55] showed that the impact of 3 T MRI noise on DPOAEs was only temporary and located in the higher spectrum of the tested frequencies (2, 6 and 8 kHz). Therefore, we would also expect this frequency range to be affected prior to the lower ones [56], which was not our case. Another source of variability in our study may be associated with the occlusion effect generated by the use of earphones during MRI testing. As with the effectiveness of the noise reduction, the importance and impact of the occlusion effect depend on the placement of the earphone inserts in the ear canal [57]. This effect increases the amount of energy reaching the tympanic membrane through bone conduction. The impact of the occlusion effect may vary depending on the specific MRI sequences used, as each sequence generates different levels of vibration and noise. This variability could have influenced our results and limited our ability to accurately interpret the data (see below for a detailed discussion on this limitation).

For the second subclinical test (extended high frequency thresholds), a subset of the participants exhibited mild and/or severe threshold increases for some of their observations, while others did not. This frequency range is known to be most sensitive to early hearing loss, and may be an indicator of possible future damage if the exposure continues [29]. Sub-05 was tested more intensively than other participants to make sure that pre-existent hearing loss was not made worse by the scanning protocol. They are the only participant that crossed the 20-dB-HL severe shift boundary [39] in one session, in connection with an anatomical scan, but they otherwise mostly showed negligible changes (≤ 5 dB HL) or even improvements. Their results allowed us to see that frequencies showing threshold increases in the extended high frequency range also happened to show threshold decreases for those frequencies throughout the different sessions, suggesting a greater test-retest variability for this range than for the standard frequencies. A greater variability could also mean that the data points that would seem to be alarming could also be the product of a statistical phenomenon linked to the important number of tested frequencies and test sessions [58]. In this situation, it is expected that some measures might deviate enough to be considered significant, even in the absence of an effect. With more measures also come more individually deviant measures, but the ratio of deviant measures to the total amount of measures is generally stable [58]. Since the scanning sessions take place in a noisy environment, even with the use of hearing protection, significant improvements in the pure-tone thresholds are not the expected outcome. This wider, statistically counterbalanced distribution of measures (with both significant threshold increases and significant threshold decreases) for the extended high frequencies supports a larger test-retest variability than for the standard frequencies.

Another factor that could have an impact on variability associated with this test is the participants’ increasing level of fatigue. When participants would go through testing (pure-tone audiometry) for possible short-term changes on top of the scan session, they would spend between 3.75 and 5.25 hours at the research center, doing all kinds of tasks in and out of the scanner. Since pure-tone audiometry is a task that requires a certain level of focus and effort, it is possible that some participants showed a drop of energy, motivation, and/or attention at some point in the process, thus impacting the results. The extended high frequencies can already be hard to detect for some people. When their attention is running low, the possibility that detection thresholds increase can also increase. Therefore, we conclude that most of the increases observed in the extended high frequencies would fall within the greater test-retest variability of this range. Because the affected frequencies were inconsistent either within or between participants, we also conclude an absence of clear loss in hearing threshold immediately after exposure to an MRI scan. Moreover, if any of the observed increases had become permanent, they should also have been consistently observed in the delayed observations.

These findings confirm the results presented by Lim et al. [59] in their study on the impact of 3 T scanner noise exposure on extended high-frequency hearing thresholds. For their study, they recruited 35 patients undergoing a variety of head and neck MRI acquisition sessions. Pure-tone thresholds up to 14 kHz were acquired before and after their MRI session, and the patients wore foam earplugs during the MRI session. They did not detect statistically significant threshold shifts for 8, 10, 12 and 14 kHz [59], which seems to align with our findings, even though their participants were scanned for shorter periods (mean = 27 minutes 52 seconds, SD = 6 minutes 40.22641 seconds, range: from 17 minutes 20 seconds to 46 minutes 50 seconds).

### Permanent changes in hearing outcomes

The second experimental condition used to evaluate the potential clinical and subclinical impact of MRI noise exposure (with hearing protection) supplied a portrait of the possible long-term impact of this exposure. For such impacts to be considered linked to the MRI noise exposure, short-term impacts should also have been observed for the same frequencies and these impacts should be present during subsequent delayed observations. The clinical pure-tone thresholds obtained during the delayed observations showed isolated and inconsistent (between ears, sessions and/or participants) differences. These few significant threshold shifts could be related to the scanner noise exposure, but could also be linked to noise exposure that occurred outside of the scanning context in the time interval between the baseline acquisition session (January–February 2021) and a specific long-term observation. Their limited number could also be an indication of a statistical phenomenon due the large number of tested frequencies and observations [58].

Similarly, subclinical long-term tests also showed inconsistent effects of the MRI noise exposure. As with short-term observations, subclinical effects were evaluated with the combination of the DPOAE amplitudes and the extended high frequency pure-tone thresholds (9 kHz– 20 kHz). The observations on long-term changes of DPOAE amplitudes mostly showed small or no deterioration of the emissions, but some participants showed great within-session variability for some sessions that was absent from other time points. It is also possible to notice that some of the participants are systematically missing values in the higher tested F2 frequencies. This last effect could in part be attributed to the normal ageing process linked with day-to-day noise exposure [50], but a part could also be linked to noise-induced (by the scanner or other noise sources) fatigue of the outer hair cells of the cochlea. Repeated deterioration profiles like the one that can be seen for sub-02 (for F2 = 8 and 10 kHz) could be an indicator that this participant might have had a time-enduring decrease of the outer hair cells of the cochlea in this specific frequency range, since the observations on short-term changes show similar missing values and the rest of their tested frequencies show results closer to or within test-retest variability.

On the opposite side, different inter-session patterns like what can be observed in sub-03’s results (see Fig 4, panel c) could be another indicator of an improper fitting of the hearing protection equipment during some of the scan sessions since notches are present at different frequencies and ear sides from the first session (ses-03) to the second session (ses-06). This type of result highlights the importance of the use and proper placement of hearing protection equipment while exposed to MRI noise. A similar phenomenon could also be present with sub-06. In one session, sub-06 showed a deterioration in DPOAE amplitudes (see Fig 4, panel c: ses-07) only for one ear, while such deterioration was completely gone at a later session (see Fig 4, panel b: ses-10), leaving DPOAE amplitudes improvements instead. Even though anatomical and functional scans do not use the same types of acquisition sequences, thus resulting in different noise exposures, there should not be such different within-participant response profiles between those two sessions if the protection equipment was properly installed for both of them.

Extended high frequency thresholds (9 kHz– 20 kHz) also showed inconsistent noise exposure effects. Threshold increases observed in the extended high frequencies were of greater magnitude (between 10 and 20 dB HL) than those observed in the conventional frequencies (250 Hz– 8 kHz; between 10 and 15 dB HL), but similarly to the short-term extended high frequency thresholds, results showed a greater test-retest variability for this frequency range. The repeated exposure to the scanner noise since the baseline data were acquired could play a role, important or not, in the threshold shifts measured during the delayed observations. On the other hand, other factors like the participants’ level of fatigue during the test session or noise exposure from other contexts than the MRI could also be sources of difference. The more frequent changes observed in the extended high frequency spectrum (> 8 kHz) are also compatible with age-related hearing thresholds shifts [60], although the duration of this study would not support this factor as the main reason for these changes [60]. Then again, just as it was possible to observe improvements in the extended high frequencies during the observations on short-term changes, observations on long-term changes also showed some improvements of the pure-tone thresholds in that frequency range. While 9, 10 and 12.5 kHz seem to be the most frequently impacted frequencies in the extended high frequency range, 9 and 10 kHz also showed improvements for some participants. Therefore, there seems to be no consistent pattern related to threshold gains or losses either between participants, sessions and/or ears in the delayed observations, supporting the inference that there is greater test-retest variability for this range of frequencies, even though prior studies showed the opposite [38, 61]. Unfortunately, those studies had limited numbers of retest sessions (Schmuziger et al. [38]: two intrasessions (one for each of the two pieces of stimuli presentation equipment used); Mishra et al. [61]: one intrasession (within 1 to 4 hours) and one intersession (within 1 to 12 days, mean = 10)), giving a limited picture of the test-retest variability of the extended high frequency pure-tone thresholds. The larger number of test sessions presented in this study could explain why the phenomenon was not observed in these previous studies. Nonetheless, losses in this frequency range are known to be linked with the ageing process of the cochlea [62], and their presence following noise exposure may be an indicator of future permanent damage. Therefore, special attention should be paid to this frequency range in any hearing conservation protocol.

When looking at the combination of the DPOAE amplitudes and extended high frequency thresholds, it is possible to conclude that the isolated amplitude decreases and rightfully significant threshold increases are more probably linked to the one-time effect of an improper equipment fit or an exposure to noise outside of the scanner’s context.

In summary, considering the absence of a consistent clinical and subclinical impact of the noise exposure on the different tests, we propose that the long-term threshold and DPOAE amplitudes shifts we observe are not likely to be directly attributed to scanning sessions’ noise, and rather that they are likely the result of a combination of elements led by the exposure to other day-to-day noise sources, isolated cases of improper hearing protection use and the large number of data points that are compared.

### Limitations

In light of the inconsistencies reported in the results section and the methodology used to acquire the data through an already ongoing research platform, it was hard to reach a definitive conclusion and it came with several limitations. As mentioned before, one of the main limitations comes from the hearing protection devices. It has been shown that if the users are not properly trained on how to use them or if they are not placed properly, their efficiency varies drastically [63, 64]. This is why Hayes et al. [63] recommend the implementation of training and fit tests for hearing protection users. Kozlowski et al. [65] brought up the idea of a simple and affordable tester to verify the placement of earplugs. These procedures could limit the variability of the placement and, therefore, limit the variability in the efficiency of the equipment used.

It was also mentioned before that pure-tone audiometry has an inherent limitation. As it is a test where participants are required to give a form of behavioral response, their levels of fatigue, attention and motivation can all have an impact on the quality and accuracy of the results it provides.

The next main limitation is linked to the enduring nature of the project. Participants are exposed to the scanner noise, but also to noise coming from other sources throughout the week. It is impossible to completely control their exposure, and keeping a record of every exposure over a timespan of multiple years would also be a great challenge. While the observations on short-term changes are mostly immune to this bias, the results from observations on long-term changes are directly impacted by the noise exposure in the period between the scan session and the hearing test session, but also by the general exposure that occurred since the baseline data were acquired.

Another important limitation mentioned above relates to the occlusion effect caused by the use of insert earphones during the MRI procedures. The occlusion effect refers to the amplification of low-frequency sounds that occurs when the outer part of the ear canal is blocked, as happens when insert earphones are used. This blockage traps sound waves inside the ear canal, leading to an increase in the perception of one’s own voice and other internal sounds. Additionally, external low-frequency noises, such as the noise and vibrations produced by an MRI machine, can be intensified due to the occlusion effect. In the context of our research, we may expect around a 20–30 dB occlusion effect boost due to the noise and vibration from the scanner [57]. This amount is similar to the attenuation of air conduction provided by the hearing protection utilized by the participants during MRI testing, although such levels may vary depending on the actual levels of noise and vibration emitted by each MRI sequence. The occlusion effect can be measured by comparing the level of sound pressure in the ear canal with and without the ear canal being occluded. This measurement typically involves using a probe microphone placed near the tympanic membrane to assess the increase in SPL when the ear canal is blocked by an insert earphone. In our study, while we provided participants with hearing protection to mitigate the loud noises generated by the MRI machine, we did not measure the degree to which the occlusion effect might have boosted the energy from the MRI noise and vibrations. This is a significant limitation as it introduces uncertainty regarding the actual noise exposure experienced by participants. The potential enhancement of low-frequency sounds due to the occlusion effect could mean that participants’ ears were subjected to higher levels of noise than we accounted for, possibly affecting our findings related to hearing changes. Future studies should consider measuring the occlusion effect to more accurately gauge the noise exposure participants receive during MRI scans. By doing so, we can better understand the potential impact on hearing and account for any amplification of sound caused by the occlusion effect, leading to more accurate and reliable results.

Finally, the last limitation is regarding the ability to generalize these findings. First, this research project primarily involved middle-aged individuals. The effect of MRI noise exposure may vary depending on individual susceptibility to noise. In particular, older adults have an increased vulnerability to loud sounds and thus may be more susceptible to noise exposure due to various factors [66]. Since we did not include older adults in this study, the results should be interpreted with caution when applied to this population. Previous case reports on older patients undergoing Vestibular Evoked Myogenic Potential (VEMP) testing, which uses high levels of noise similar to MRI, have shown hearing losses induced by VEMP sound stimulation [66, 67]. Second, as this research project is part of a larger intensive research platform, the number of participants willing to commit to weekly MRI scans and regular auditory tests was limited. Consequently, not all participants could be tested under all experimental conditions for a sufficient amount of time to provide enough power to generalize these results properly.

Despite these limitations, this study offers a unique opportunity to study the impact of MRI noise with an unprecedented amount of exposure.

### Implications and future work

In light of the observations made possible by this research protocol, the participants of the Courtois NeuroMod project will continue their scanning regimen, and their hearing is still going to be tested, although at a less intensive rate, to ensure their continued welfare. To address the possibility of occasional improper use of the hearing protection devices, participants were given training to ensure they all use them adequately. The MRI operators will also be trained to pay attention to the proper placement of protective equipment. We also plan to evaluate the occlusion effect created in the ear canal in connection with the use of hearing protection and to quantify the level of protection granted by our hearing protection combination. The CNeuroMod project being an intensive research initiative, the relative absence of change observed in this research project suggests that patients going through single, shorter scan sessions are very unlikely to suffer adverse consequences. In a broader view, hearing research using pure-tone audiometry would greatly benefit from a clearer, per-frequency test-retest variability criteria, especially in the extended high frequencies where no clear guideline is currently available.

## Conclusion

In conclusion, the results of our study indicate that the Courtois NeuroMod project is not likely to cause hearing damage to its research participants. Although these results might not be able to be generalized to other contexts, they still provide a portrait of this specific situation to other researchers interested in deep and dense dataset projects as well as to clinicians using MRI. The importance of proper hearing protection use and placement linked to the intensity and duration of the exposure is also reiterated.

## Supporting information

S1 Fig Sub-05 hearing impairment description. This participant shows, for the left ear, a mostly flat audiometric configuration, with a moderate degree (30 to 40 dB HL) of hearing loss for the 0.25–6 kHz range, while the right ear shows normal hearing thresholds for the 0.25–8 kHz frequency range.
(DOCX)

S1 Table Pure-tone’s significant-threshold-shift criteria comparison. Comparison between different pure-tone significant-threshold-shift criteria mentioned in Methods’ Pure-tone audiometry section.
(DOCX)
