# Supplementary material for: MRI noise and auditory health: Can one hundred scans be linked to hearing loss? The case of the Courtois NeuroMod project
Source: PLoS One. 2025 Jan 17;20(1):e0309513. doi: 10.1371/journal.pone.0309513 (PMC11741633; doi:10.1371/journal.pone.0309513)
Supplement: S1 Fig — This participant shows, for the left ear, a mostly flat audiometric configuration, with a moderate degree (30 to 40 dB HL) of hearing loss for the 0.25–6 kHz range, while the right ear shows normal hearing thresholds for the 0.25–8 kHz frequency range. (DOCX) [file pone.0309513.s001.docx]

**S1 Fig - Sub-05 hearing impairment description**

This participant shows, for the left ear, a mostly flat audiometric configuration, with a moderate degree (30 to 40 dB HL) of hearing loss for the 0.25 – 6 kHz range, while the right ear shows normal hearing thresholds for the 0.25 – 8 kHz frequency range.


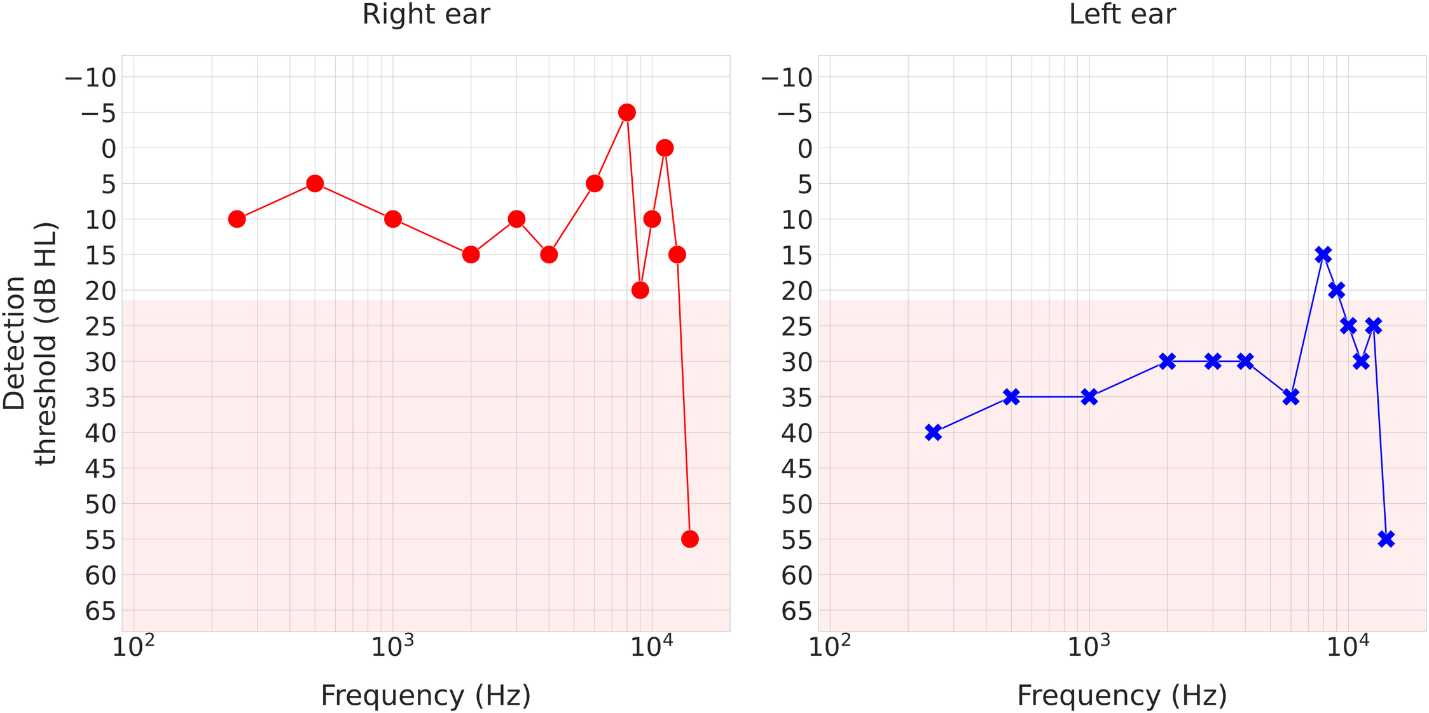


**Suppl. Fig 1. Sub-05’s baseline audiogram.** Results are presented for the right ear (left panel) and the left ear (right panel). Pure-tone threshold results are presented for both standard (0.25, 0.5, 1, 2, 3, 4, 6 and 8 kHz) and extended high frequency (9, 10, 11.2, 12.5, 14, 16, 18, and 20 kHz) ranges. A missing value indicates that the participant did not give any behavioral response at that frequency. Detection thresholds associated with hearing loss (i.e., hearing thresholds poorer than 20 dB HL [27, 28]) are highlighted with a red background.
