# Supplementary material for: MRI noise and auditory health: Can one hundred scans be linked to hearing loss? The case of the Courtois NeuroMod project
Source: PLoS One. 2025 Jan 17;20(1):e0309513. doi: 10.1371/journal.pone.0309513 (PMC11741633; doi:10.1371/journal.pone.0309513)
Supplement: S1 Table — Comparison between different pure-tone significant-threshold-shift criteria mentioned in Methods’ Pure-tone audiometry section. (DOCX) [file pone.0309513.s002.docx]

| **Participant** | **Observation**  **type** | **Frequency range** |  | **Criteria** | | | | | | | | |
| --- | --- | --- | --- | --- | --- | --- | --- | --- | --- | --- | --- | --- |
|  |  |  |  | Our criteria | ASHA,  1994 [40] | Atherley &  Dingwall-Fordyce,  1963 [47] | Dobie,  1983 [42] | Fausti et  al., 1992 [39] | Landry &  Green, 1999 [48] | NIOSH,  1998 [20] | Occ. noise exp.,  2021 [44] | Schmuziger  et al., 2004 [38] |
| sub-01 | short-term | standard |  | **No** | **No** | **No** | **No** | **No** | **No** | **No** | **No** | **No** |
|  |  | extended |  | Yes (Mild) | No | N/A | N/A | No | N/A | N/A | N/A | No |
|  | long-term | standard |  | **No** | **No** | **No** | **No** | **No** | **No** | **No** | **No** | **No** |
|  |  | extended |  | **Yes (Mild and Severe)** | **Yes** | N/A | N/A | **Yes** | N/A | N/A | N/A | **Yes** |
| sub-02 | short-term | standard |  | **No** | **No** | **No** | **No** | **No** | **No** | **No** | **No** | **No** |
|  |  | extended |  | **No** | **No** | N/A | N/A | **No** | N/A | N/A | N/A | **No** |
|  | long-term | standard |  | **Yes (Mild)** | **Yes** | **Yes** | No | **Yes** | **Yes** | No | No | **Yes** |
|  |  | extended |  | **Yes (Mild and Severe)** | **Yes** | N/A | N/A | **Yes** | N/A | N/A | N/A | **Yes** |
| sub-03 | short-term | standard |  | **No** | **No** | **No** | **No** | **No** | **No** | **No** | **No** | **No** |
|  |  | extended |  | **No** | **No** | N/A | N/A | **No** | N/A | N/A | N/A | **No** |
|  | long-term | standard |  | **Yes (Mild)** | No | **Yes** | No | No | No | No | No | No |
|  |  | extended |  | **Yes (Mild and Severe)** | **Yes** | N/A | N/A | No | N/A | N/A | N/A | **Yes** |
| sub-04 | short-term | standard |  | **No** | **No** | **No** | **No** | **No** | **No** | **No** | **No** | **No** |
|  |  | extended |  | **No** | **No** | N/A | N/A | **No** | N/A | N/A | N/A | **No** |
| sub-05 | short-term | standard |  | **No** | **No** | **No** | **No** | **No** | **No** | **No** | **No** | **No** |
|  |  | extended |  | **Yes (Mild and Severe)** | **Yes** | N/A | N/A | **Yes** | N/A | N/A | N/A | **Yes** |
|  | long-term | standard |  | **Yes (Mild)** | No | **Yes** | No | No | No | No | No | No |
|  |  | extended |  | **No** | **No** | N/A | N/A | **No** | N/A | N/A | N/A | **No** |
| sub-06 | short-term | standard |  | **No** | **No** | **No** | **No** | **No** | **No** | **No** | **No** | **No** |
|  |  | extended |  | Yes (Mild) | No | N/A | N/A | No | N/A | N/A | N/A | No |
|  | long-term | standard |  | **Yes (Mild)** | No | **Yes** | No | No | No | No | No | No |
|  |  | extended |  | **Yes (Mild)** | **Yes** | N/A | N/A | **Yes** | N/A | N/A | N/A | **Yes** |

### **S2 Table - Pure-tone’s significant-threshold-shift criteria comparison**

Occ. noise exp.: Occupational noise exposure (2021) [44]. N/A: Criteria not available or specified.
